# Overcoming resistance to STING agonist therapy to incite durable protective antitumor immunity

**DOI:** 10.1136/jitc-2020-001182

**Published:** 2020-08-26

**Authors:** Henrique Lemos, Rong Ou, Caroline McCardle, Yijun Lin, Jessica Calver, Jack Minett, Ahmed Chadli, Lei Huang, Andrew L Mellor

**Affiliations:** 1 Translational and Clinical Research Institute, Faculty of Medical Sciences, Newcastle University, Newcastle upon Tyne, Tyne and Wear, UK; 2 Georgia Cancer Center, Augusta University Medical College of Georgia, Augusta, Georgia, USA

**Keywords:** immune tolerance, immunotherapy, drug therapy, combination

## Abstract

**Background:**

Activating the Stimulator of Interferon Genes (STING) adaptor incites antitumor immunity against immunogenic tumors in mice, prompting clinical trials to test STING activators. However, STING signaling in the tumor microenvironment (TME) during development of Lewis lung carcinoma (LLC) suppresses antitumor immunity to promote tumor growth. We hypothesized that local immune balance favoring suppression of antitumor immunity also attenuates antitumor responses following STING activation. The purpose of this study was to evaluate how STING activation impacts antitumor responses in mice bearing LLC tumors.

**Methods:**

Mice bearing established LLC tumors were treated with synthetic cyclic diadenyl monophosphate (CDA) to activate STING. Mice were monitored to assess LLC tumor growth, survival and protective antitumor immunity. Transcriptional and metabolic analyses were used to identify pathways responsive to CDA, and mice were co-treated with CDA and drugs that disrupt these pathways.

**Results:**

CDA slowed LLC tumor growth but most CDA-treated mice (77%) succumbed to tumor growth. No evidence of tumor relapse was found in surviving CDA-treated mice at experimental end points but mice were not immune to LLC challenge. CDA induced rapid increase in immune regulatory pathways involving programmed death-1 (PD-1), indoleamine 2,3 dioxygenase (IDO) and cyclooxygenase-2 (COX2) in the TME. PD-1 blockade enhanced antitumor responses to CDA and increased mouse survival but mice did not eliminate primary tumor burdens. Two IDO inhibitor drugs had little or no beneficial effects on antitumor responses to CDA. A third IDO inhibitor drug synergized with CDA to enhance tumor control and survival but mice did not eliminate primary tumor burdens. In contrast, co-treatments with CDA and the COX2-selective inhibitor celecoxib controlled tumor growth, leading to uniform survival without relapse, and mice acquired resistance to LLC re-challenge and growth of distal tumors not exposed directly to CDA. Thus, mice co-treated with CDA and celecoxib acquired stable and systemic antitumor immunity.

**Conclusions:**

STING activation incites potent antitumor responses and boosts local immune regulation to attenuate antitumor responses. Blocking STING-responsive regulatory pathways synergizes with CDA to enhance antitumor responses, particularly COX2 inhibition. Thus, therapy-induced resistance to STING may necessitate co-treatments to disrupt regulatory pathways responsive to STING in patients with cancer.

## Introduction

During tumor development local inflammation establishes immune checkpoints (ICPs) that suppress antitumor immunity in primary lesions and tumor-draining lymph nodes (TDLNs), which together constitute the immune tumor microenvironment (TME). Blocking cytotoxic T-lymphocyte-associated protein-4 (CTLA-4) and programmed cell death-1 (PD-1) interactions that contribute to ICPs generated promising clinical responses in some cancers.^1^ PD-1 blockade is effective in up to 45% of patients with melanoma but is far less effective in other cancers, with ~20% response rates typical in patients with non-small cell lung carcinoma (NSCLC).^2 3^ Many factors may contribute to differential responses to ICP blockade including heterogeneity in patient genetics, immune status and treatment history, plus variations in the inflammatory and immunological landscapes of specific cancer types. In mouse tumor models, tumor immunogenicity, reflecting differential immune cell infiltration in the TME, correlates with responsiveness to PD-1 blockade.^4^ While immunogenic (hot) tumors such as B16 melanomas transfected to express neo-antigens are responsive to PD-1 blockade, weakly immunogenic (cold) Lewis lung carcinoma (LLC) tumors are refractory to PD-1 blockade.^2–5^ Thus, the LLC model recapitulates the poor clinical responses to PD-1 blockade observed in most patients with lung cancer.

Resistance to PD-1 blockade (nivolumab) therapy in patients with advanced melanoma and renal cell carcinoma correlated with elevated oxidative tryptophan metabolism mediated by indoleamine 2,3 dioxygenase (IDO) following PD-1 blockade, suggesting that adaptive therapy resistance may be a barrier to inciting effective and durable clinical responses to immunotherapy.^6^ Poor patient survival after radiotherapy/chemotherapy (RT/CT) also correlated with elevated levels of systemic IDO activity,^7 8^ implicating IDO as a potential barrier to durable antitumor responses after RT/CT. However, monotherapy to inhibit IDO is ineffective in most patients with cancer and the IDO inhibitor epacadostat did not improve survival of patient with melanoma when combined with PD-1 blockade, raising questions about the role of IDO in resistance to therapy and the efficacy of IDO inhibitor drugs in patients with cancer.^9 10^ Nevertheless, regulatory pathways induced during tumor development (intrinsic) and/or responsive to therapy (adaptive) may mediate and potentiate resistance to therapy, respectively. Intrinsic and adaptive therapy resistance may be influenced by tumor type, size and immunogenicity, and patient immune status. Regulatory pathway heterogeneity and redundancy may also impact the potency and plasticity of ICPs before and after therapy.

Direct introduction of innate immune adjuvants into the TME is an alternative strategy to boost antitumor immunity. Intratumoral treatment with a synthetic cyclic diadenyl monophosphate (CDA) derivative to activate the signaling adaptor *Stimulator of Interferon Genes* (STING) led to long-term tumor-free survival of mice with relatively immunogenic tumors such as B16 melanoma.^11^ CDA treatment induced dendritic cells (DCs) to release interferon type I (IFN-I) and cross-present antigens to activate effector CD8 T cells. Previously, we reported that STING/IFN-I signaling promoted LLC tumor growth due to increased local immune regulation mediated by TDLN DCs expressing IDO.^12^ In the current study, we tested if direct CDA treatment controlled growth of LLC tumors. CDA treatment slowed growth of established LLC tumors and prolonged survival, but most mice still succumbed to primary tumor growth and CDA treatment did not induce surviving mice to acquire stable protective antitumor immunity. Direct CDA treatment stimulated rapid elevation of multiple immune regulatory pathways involving PD-1, IDO and COX2 in the TME. Disrupting each pathway independently enhanced antitumor responses to CDA, particularly co-treatment with a COX2 inhibitor drug. Thus, the intrinsic plasticity of physiological responses to immunotherapy means that monotherapies to disrupt ICPs or activate STING are relatively ineffective, while co-treatments that activate STING and disrupt pathways contributing to ICPs boost antitumor responses by reducing intrinsic and adaptive resistance to STING agonists.

## Results

### Established LLC tumors are resistant to the antitumor effects of direct STING activation

**Figure 1:**
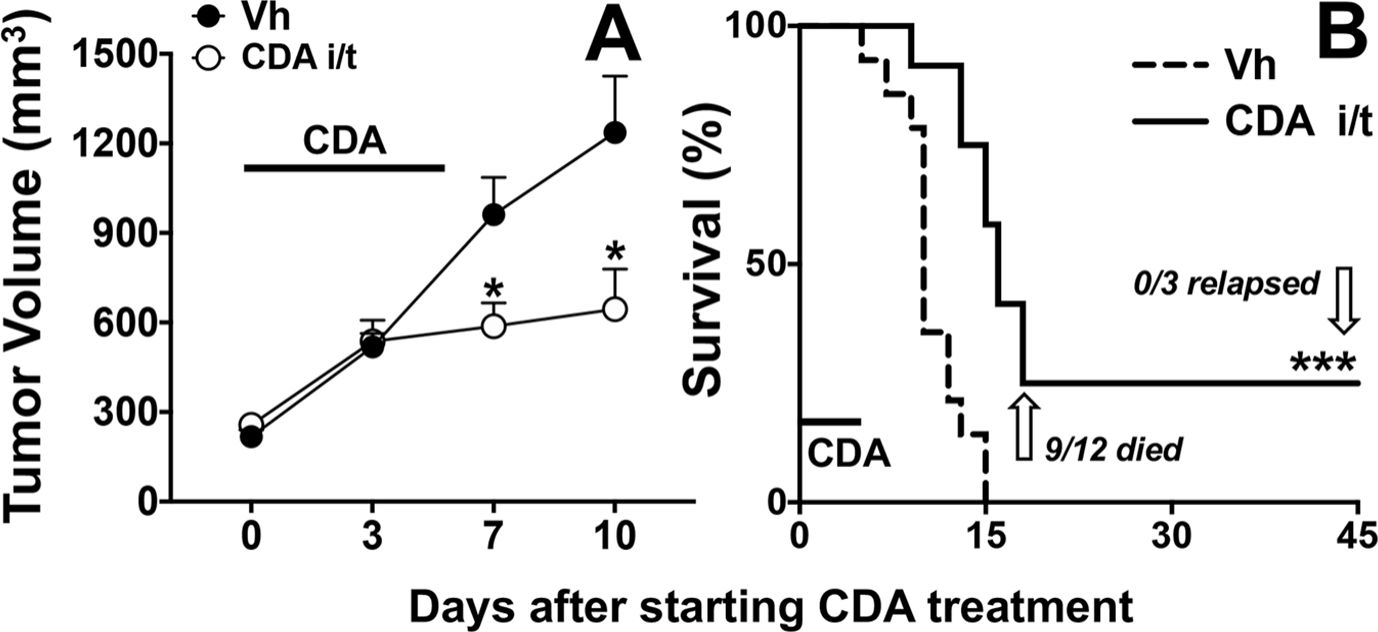
Cyclic diadenyl monophosphate (CDA) treatment to activate Stimulator of Interferon Genes controls Lewis lung carcinoma (LLC) tumor growth transiently. B6 mice with established (250–350 mm^3^) LLC tumors were treated with CDA (100 µg/mouse, intratumorally, days 0, 2, 6). Tumor volumes (A) and mouse survival (B) were scored until experimental end points, when all mice were examined to assess if primary tumor burdens were absent (tumor clearance), or if residual tumor tissues and/or distal metastatic tumors were present (tumor relapse). Data (mean±SEM) were analyzed using two-way analysis of variance with Bonferroni’s multiple comparisons test for each time point (A) or log-rank test (B), n=12. *P<0.05, ***p<0.001. Vh, vehicle.

B6 mice were inoculated with LLC tumor cells and when dermal tumors were well-established (250–300 mm^3^) mice were given a short course of direct (intratumoral) CDA treatment (100 µg on days 0, 2, 6). CDA treatment did not slow tumor growth immediately (day 3) but after the final CDA treatment tumor growth was reduced significantly (figure 1A). However, tumor control was transient as growth resumed in most mice, and most CDA-treated mice (75%) had to be sacrificed by 15 days after CDA treatment was initiated due to excessive tumor growth, extending survival by only ~5 days more than control mice treated with vehicle (figure 1B). CDA-treated mice that did not succumb to tumor growth (~25%) were challenged with a second bolus of LLC cells on the contralateral flank when primary lesions had healed, 60 days after CDA treatment was initiated. Secondary LLC tumors grew at the same rates as primary tumors in all mice (table 1, group B), indicating that direct CDA treatment did not incite stable protective antitumor immunity, even though primary tumors were eliminated by CDA monotherapy.

**Table 1:** CDA reinforces immune regulation to attenuate antitumor responses

Group	CDA	Co-treatment (with CDA)			Induced ICP pathways			Outcomes		
								Survival*	Relapse†	Immunity
		αPD-1	IDOi	COX2i	PD-L1	IDO	COX2			
A	–							0/46	–	
B	+				+	+	+	11/47	0/11	0/11‡
C	+	**+**				+	+	15/15	15/15	–
D1 D2 D3	+ + +		1MT NLG BMS		+ + +		+ + +	5/8 4/8 14/14	0/2 0/4 14/14	–
E	+	+	BMS					8/9	8/8	–
F1 F2	+ +			+ +	+ +	- -		15/15 8/9	0/15 0/8	15/15‡ 8/8§
G	+	*Small LLC tumors (~100 mm^3^)*						15/15	0/15	15/15‡

Numbers represent total number of mice from combined experiments.

*No. of mice surviving to end point (>60 days).

†No. of mice with tumors at end points (day 60 for groups A-F1, day 30 for group F2).

‡No. of mice resistant to LLC re-challenge at day 60.

§No. of mice resistant to primary and distal tumor growth (abscopal effects).

CDA, cyclic diadenyl monophosphate; COX2i, cyclooxygenase-2 inhibitor; ICP, immune checkpoint; IDOi, indoleamine 2,3 dioxygenase inhibitor; LLC, Lewis lung carcinoma; PD-1, programmed death-1; PD-L1, programmed death ligand-1.

In a previous study, CDA treatment to activate STING protected 50% of mice implanted with immunogenic B16 melanomas until experimental end points and surviving mice all acquired stable protective antitumor immunity.^11^ As CDA treatment was initiated when B16 tumors were relatively small (~100 mm^3^) in this prior study, we next tested if CDA treatment was more effective in mice with smaller LLC tumors. Mice were treated with CDA as before, except that treatment was initiated when LLC tumors were first detected (90–120 mm^3^), 4–5 days earlier than in our initial study on mice with more established tumors. Earlier CDA treatment prevented progression of small LLC tumors, leading to rapid tumor regression and uniform survival (online supplementary figure S1A, B). In addition, earlier CDA treatment induced all mice to acquire stable protective immunity to LLC challenge (table 1, group G). Direct CDA treatment was necessary to control LLC tumor growth as intravenous CDA treatment when tumors first appeared had little impact on tumor growth and had no significant effect on survival (online supplementary figure S1C, D). Direct CDA treatment did not protect STING-deficient (STING-KO) mice with small LLC tumors (online supplementary figure S2), confirming that therapeutic responses were STING-dependent and revealing that STING expressed by LLC tumor cells was not the relevant therapeutic target of CDA.

10.1136/jitc-2020-001182.supp1 Supplementary data

### Direct CDA treatment stimulates multiple immune regulatory pathways in the TME

**Figure 2:**
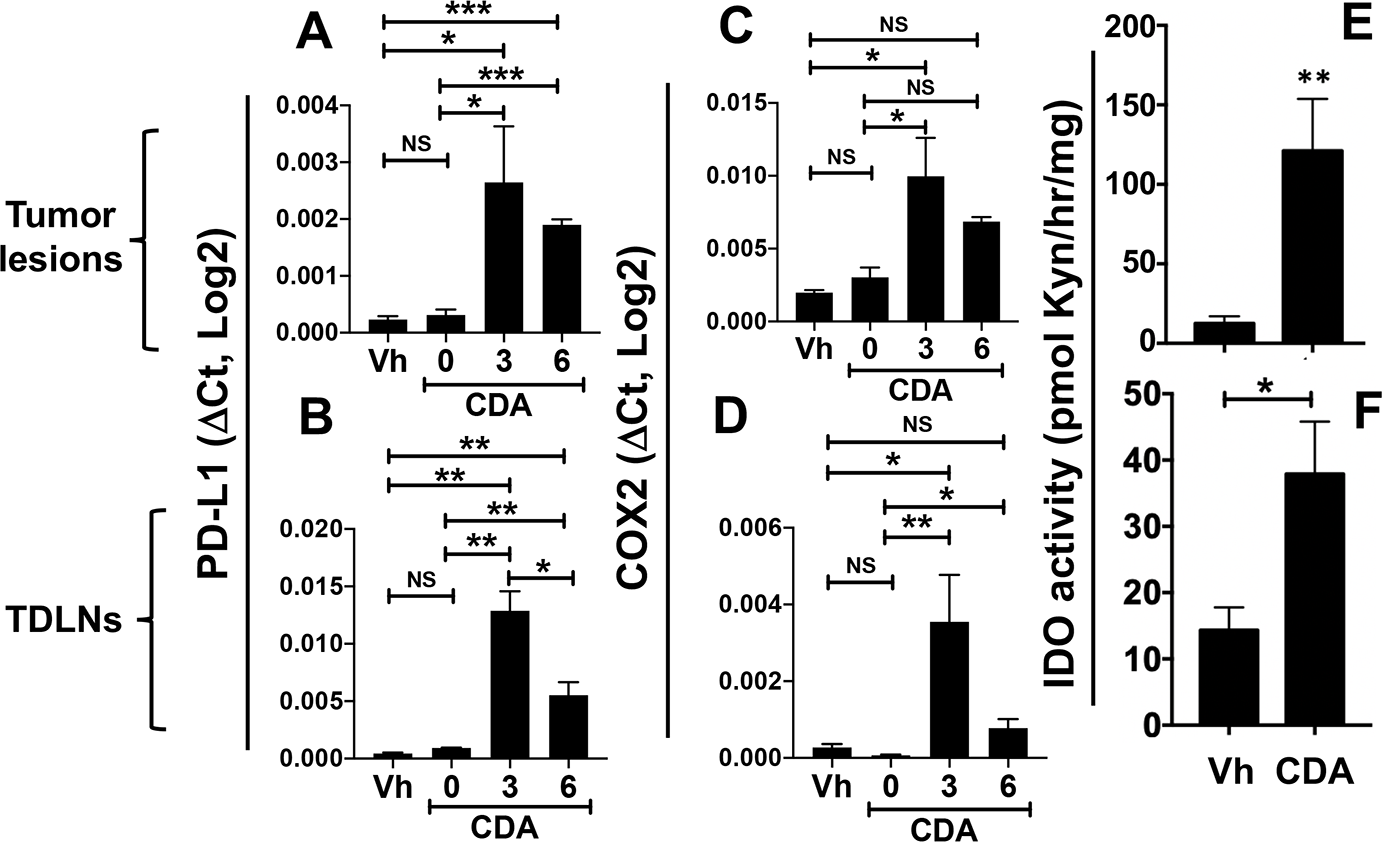
Direct cyclic diadenyl monophosphate (CDA) treatment enhances immune regulatory pathways in the tumor microenvironment (TME). (A–D) Mice with established Lewis lung carcinoma (LLC) tumors (250–350 mm^3^) were treated with CDA twice, sacrificed at the times indicated after the final CDA treatment (0 hour), and programmed death ligand-1 (PD-L1) and cyclooxygenase-2 (COX2) gene transcription in tumor lesions (A, C) and tumor-draining lymph nodes (TDLNs) (B, D) was assessed by qRT-PCR. Vehicle (Vh)-treated mice were sacrificed 3 hours after treatment. (E, F) Mice with established LLC tumors were treated with CDA twice and 24 hours later tumor lesion (E) and TDLN (F) tissues were homogenized to assess indoleamine 2,3 dioxygenase (IDO) activity (see ‘Methods’ section). Data (mean±SEM) were analyzed using Mann-Whitney U tests, n=3–6. *P<0.05, **p<0.01, ***p<0.001. NS, not significant.

As resistance to the antitumor effects of CDA developed rapidly as tumors became more established, we assessed if CDA treatment induced immune regulatory pathways that commonly contribute to ICPs and impede antitumor immunity, as potential causes of therapy resistance. Cells expressing programmed death ligand-1 (PD-L1) induce T cells expressing PD-1 to become functionally exhausted and/or undergo apoptosis. Like many patients with cancer, mice with LLC tumors are poorly responsive to blockade of PD-1/PD-L1 signaling.^2–5^ Direct CDA treatment induced rapid and significant increase in PD-L1 gene transcription in tumor lesions (figure 2A) and TDLNs (figure 2B), and elevated PD-L1 expression was sustained for at least 6 hours in both TME sites. COX2 expression increases in the TME during LLC tumor growth and COX2 inhibitors slow LLC tumor growth.^13 14^ Direct CDA treatment potentiated COX2 gene transcription rapidly in tumor lesions and TDLNs, and elevated COX2 transcription persisted for over 6 hours in both TME sites (figure 2C, D).

Next, we tested if direct CDA treatment induced IDO enzyme activity in tumor lesions and TDLNs by assessing production of kynurenine (Kyn), a tryptophan catabolite made by cells expressing IDO, in TME tissues from mice with LLC tumors. LLC tumor cells do not express IDO but IDO activity induced in TDLN DCs via STING/IFN-I signaling during LLC tumor growth suppresses antitumor immunity to promote optimal LLC tumor growth.^12^ STING agonists also induced DCs to express IDO, an attribute we exploited to attenuate autoimmune disease in mouse models of rheumatoid arthritis, multiple sclerosis and type I diabetes.^15–19^ Direct CDA treatment potentiated IDO activity rapidly and significantly in tumor lesions and TDLNs, relative to levels induced by LLC growth (figure 2E, F), Thus, intratumoral STING activation induced rapid increase in local expression of PD-L1, COX2 and IDO, which may attenuate the antitumor attributes of CDA.

### PD-1 blockade enhances antitumor responses to CDA

**Figure 3:**
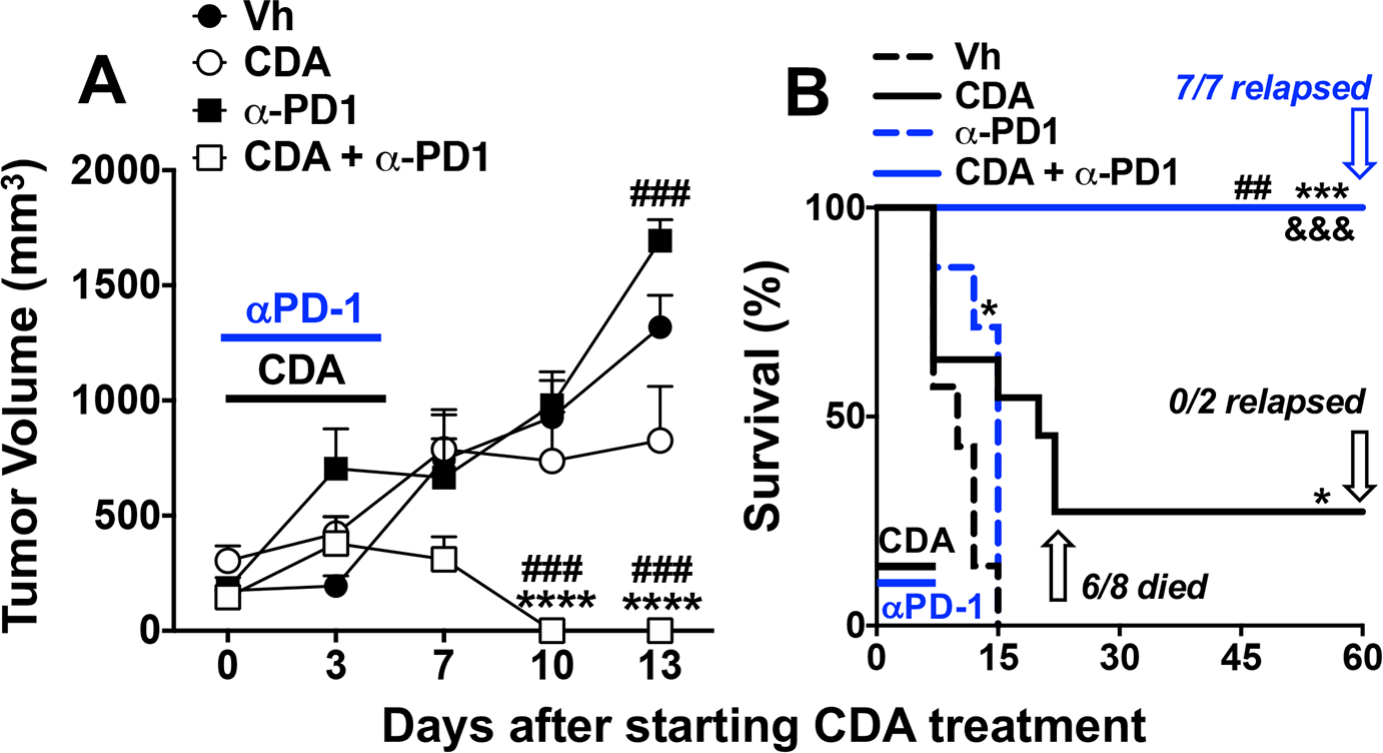
Programmed death-1 (PD-1) blockade enhances antitumor responses to cyclic diadenyl monophosphate (CDA). (A, B) B6 mice with established Lewis lung carcinoma (LLC) tumors were treated with CDA with or without anti-PD-1 monoclonal antibodies (mAbs) to disrupt immune checkpoints (ICPs) (see ‘Methods’ section). Tumor volumes (A) and mouse survival (B) were assessed until experimental end points (day 60). At end points mice were examined to assess tumor clearance or relapse. Data (mean±SEM) were analyzed using two-way analysis of variance with Bonferroni’s multiple comparisons test for each time point (A) or the log-rank test (B), n=7–9. *P<0.05, ***p<0.001, ****p<0.0001 (vs vehicle (Vh)); ##p<0.01, ###p<0.001 (vs CDA monotherapy); &&& p<0.001 (vs anti-PD1 monotherapy).

To test if CDA-induced PD-L1 expression reinforces ICP potency mice were co-treated with CDA and anti-PD-1 monoclonal antibodies (mAbs) to block PD-L1/PD-1 interactions that inactivate effector T cells. Consistent with LLC tumors being refractory to PD-1 blockade, anti-PD-1 mAb monotherapy (150 µg/mouse, intraperitoneal, 15 min before each CDA injection) did not slow LLC tumor growth (figure 3A), and had minimal survival impact (figure 3B). As before (figure 1), direct CDA monotherapy did not prevent tumor growth in most mice (75%). Combining direct CDA treatment with PD-1 blockade led to rapid and uniform tumor regression and uniform survival until experimental end points (figure 3). No overt evidence of tumor relapse was observed in any mice co-treated with CDA and anti-PD-1 mAbs but some mice became moribund at day 60 prompting their sacrifice. On examination, all mice that became moribund had invasive and dispersed tumors originating at primary lesions and expanding into the peritoneal cavity (ie, they were not visible externally), and some mice also had distal (metastatic) tumors growing in lungs and/or liver (table 1, group C). These observations prompted sacrifice of remaining mice in this treatment group and on inspection all mice had invasive internal tumors and some had metastatic tumors. Thus, combining CDA and PD-1 blockade enhanced tumor control and survival but did not eliminate primary tumor burdens, indicating that induced antitumor immunity was transient and unstable.

### IDO inhibition enhances antitumor responses to CDA

**Figure 4:**
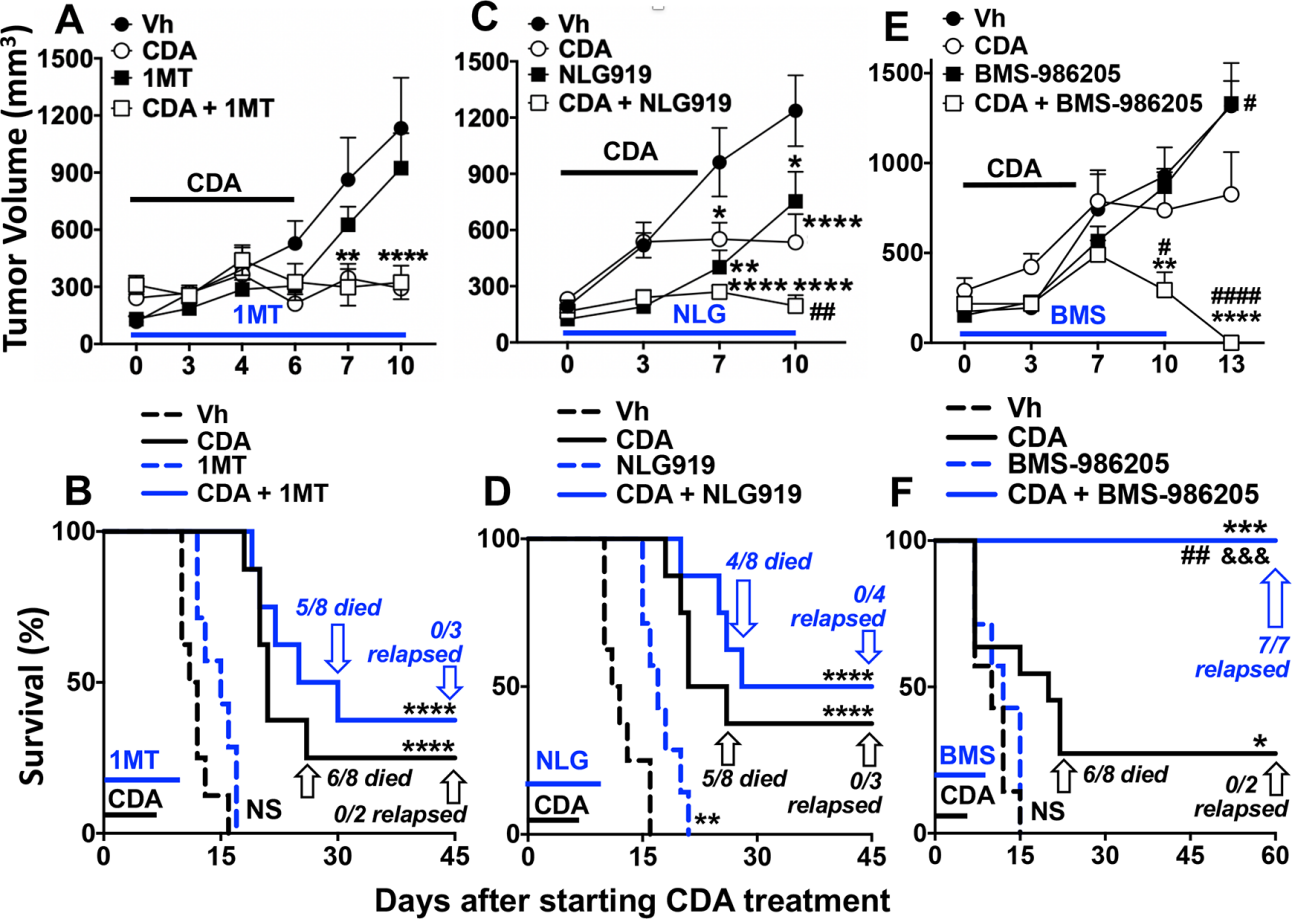
Indoleamine 2,3 dioxygenase (IDO) inhibition enhances antitumor responses to cyclic diadenyl monophosphate (CDA). (A–F) B6 mice with established Lewis lung carcinoma (LLC) tumors were treated with CDA with or without IDO inhibitors, 1MT (A, B), NLG-919 (C, D) and BMS-986205 (E, F). Tumor volumes (upper panels) and survival (lower panels) were assessed until experimental endpoints. At end points mice were examined to assess tumor clearance or relapse. Data (mean±SEM) were analyzed using two-way analysis of variance with Bonferroni’s multiple comparisons test for each time point (A, C, E) or log-rank test (B, D, F), n=7–9. *P<0.05, **p<0.01, ****p<0.0001 (vs vehicle (Vh)); #p<0.05, ##p<0.01, ####p<0.0001 (vs CDA monotherapy); &&& p<0.001 (vs BMS-986205 monotherapy).

Next, we tested if three different proprietary IDO inhibitor (IDOi) drugs with distinct pharmacological characteristics enhanced therapeutic responses to direct CDA treatment in the LLC tumor model. Indoximod (1-methyl-D-tryptophan (1MT)), navoximod (NLG-919) and lindrostat (BMS-986205) have all been tested in patients with cancer as monotherapies, or in combination with other anticancer treatments, with varying effects on clinical outcomes.^10^ Pharmacologically, lindrostat is the most effective IDO inhibitor, although many factors may impact the antitumor attributes of IDOi drugs.^10^ Mice with established LLC tumors were given CDA or IDOi monotherapy, or co-treated with CDA and an IDOi drug. As before, direct CDA monotherapy prevented tumor growth in only 20%–30% of mice (figure 4). Monotherapies with 1MT (2 mg/mL in drinking water days 1–10) or lindrostat (2 mg/kg, daily gavage, days 0–10) had no significant effects on tumor growth (figure 4A, E) or survival (figure 4B, F), relative to vehicle-treated mice. Navoximod (intraperitoneal, 5 mg/kg, daily from days 1–10) monotherapy slowed tumor growth and increased survival by 5–6 days (figure 4C, D).

Co-treatment with 1MT did not enhance CDA-mediated tumor control or increase survival significantly, relative to outcomes with CDA monotherapy (figure 4A, B). Navoximod synergized with CDA to further reduce tumor growth but did not increase survival significantly, relative to CDA monotherapy (figure 4C, D). No residual primary or metastatic tumors were found in mice surviving to experimental end points after co-treatments with CDA and 1MT or navoximod (figure 4B and D, table 1, groups D1, D2), as in mice given CDA monotherapy. In contrast, lindrostat co-treatment enhanced antitumor responses to CDA significantly, promoting rapid tumor regression and uniform survival to experimental end points (figure 4E, F). Nevertheless, on inspection at experimental end points (day 60) all mice in this treatment group had invasive tumors originating at primary lesions growing into the peritoneal cavity, and some mice also had metastatic tumors in lungs and liver (table 1, group D3). Thus, as in mice co-treated with CDA and PD-1 blockade, CDA and lindrostat co-treatment induced rapid tumor regression but did not eliminate primary tumor burdens, leading to tumor relapse at primary and distal sites. Collectively, our findings reveal that increased IDO activity in the TME due to STING activation attenuates antitumor responses to CDA, and lindrostat was the only one of three proprietary IDOi drugs tested that synergized with CDA to enhance antitumor responses substantively.

DCs expressing IDO in the TME suppress effector T cells and activate regulatory CD4 T cells (Tregs) to attenuate T cell immunity via PD-1-dependent mechanisms.^20^ To investigate potential links between these T cell regulatory pathways, we assessed if PD-1 blockade or IDOi drugs impacted IDO activity and PD-L1 expression induced by CDA treatment. PD-1 blockade had no effect on CDA-induced IDO activity in tumor lesions or TDLNs (online supplementary figure S3A, B). Lindrostat (BMS) co-treatment partially reduced CDA-induced PD-L1 expression in tumor lesions but had no significant impact on CDA-induced PD-L1 expression in TDLNs (online supplementary figure S3C, D). Thus, the IDO and PD-1 pathways make non-redundant contributions that promote resistance to STING activation independently, as disrupting either pathway enhanced antitumor responses to CDA. Since co-treatments to activated STING and block PD-1 or inhibit IDO led to eventual tumor relapse, we tested if simultaneous PD-1 and IDO blockade improved tumor control. Lindrostat plus PD-1 blockade co-treatment did not enhance tumor control or increase survival, relative to outcomes in vehicle-treated mice (online supplementary figure S3E, F), reflecting outcomes reported for the ECHO-301 clinical trial using epacadostat to inhibit IDO activity.^9^ As expected, combined lindrostat and PD-1 blockade enhanced tumor control and prolonged survival after CDA treatment without dermal tumor relapse (online supplementary figure S3E, F). However, on inspection at experimental end points all mice in this treatment group had invasive tumors originating from primary lesions growing into the peritoneal cavity, and some mice also had metastatic tumors (table 1, group E). Thus, simultaneous disruption of the PD-1 and IDO regulatory pathways did not improve antitumor responses to CDA, as tumor relapse still occurred in all mice given such treatments.

### COX2 inhibition and STING activation synergize to induce protective antitumor immunity

**Figure 5:**
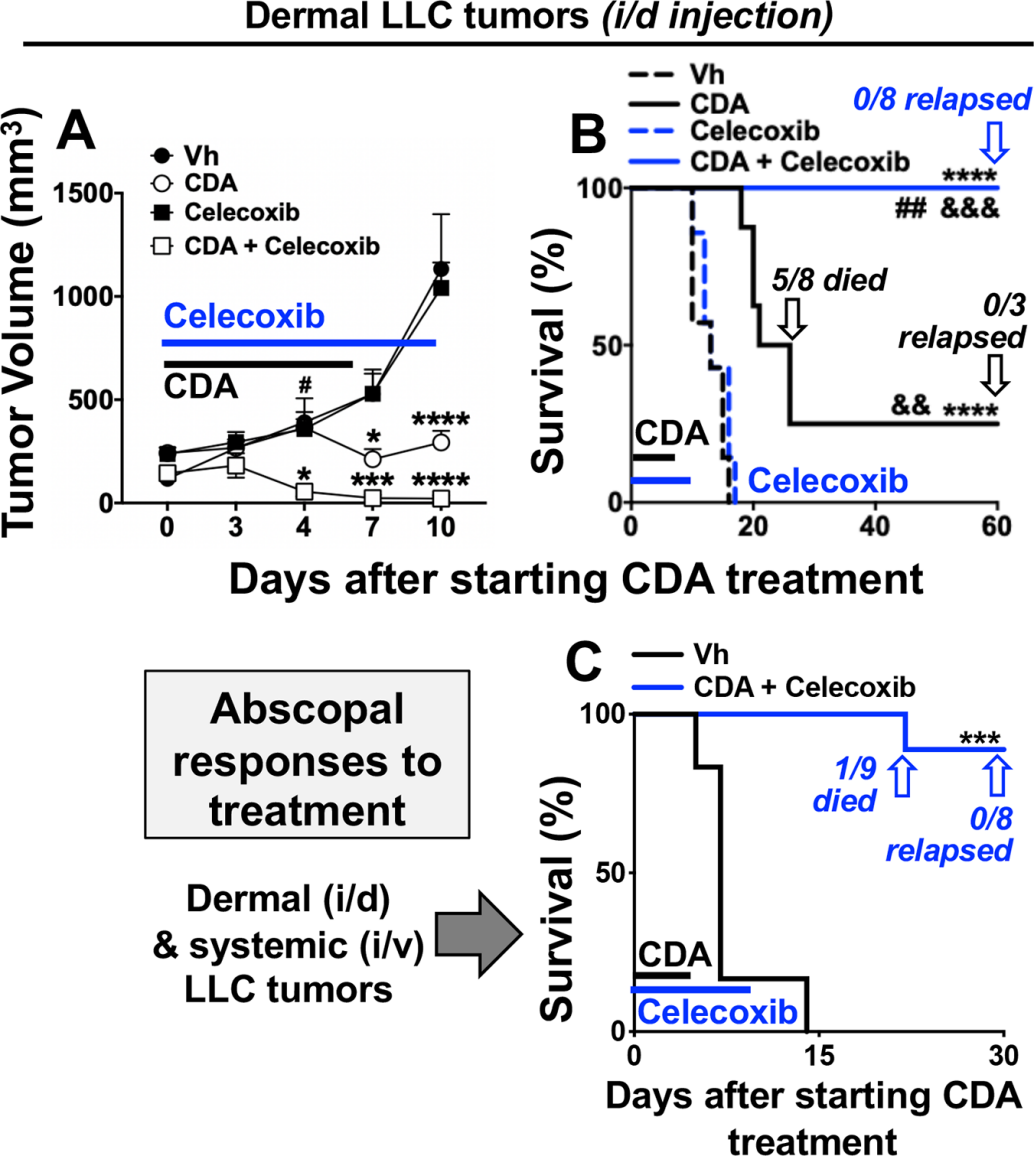
Celecoxib and cyclic diadenyl monophosphate (CDA) co-treatments induce protective antitumor immunity. (A, B) B6 mice with Lewis lung carcinoma (LLC) tumors were treated with CDA with or without celecoxib (60 mg/kg, oral gavage) and tumor volumes (A) and mouse survival (B) were assessed. At end points mice were examined to assess tumor clearance or relapse. (C) Mice were injected with LLC cells on day 0 (2×10^5^ cells/mouse, intradermal (i/d)) and again on day 5 (5×10^5^ cells/mouse, intravenous (i/v)) and were treated as indicated. Data (mean±SEM) were analyzed using two-way analysis of variance with Bonferroni’s multiple comparisons test for each time point (A) or log-rank test (B, C), n=7–9. *P<0.05, **p<0.01, ***p<0.001, ****p<0.0001 (vs vehicle (Vh)); ##p<0.01 (vs CDA monotherapy). &&P<0.01, &&&p<0.001, &&&&p<0.0001 (vs celecoxib monotherapy).

Next, we tested if a COX2-selective inhibitor, celecoxib, boosted antitumor responses to CDA. Mice with established LLC tumors were co-treated with CDA (dosing as before) and celecoxib (60 mg/kg, oral gavage, days 0–10). Celecoxib monotherapy had no effect on tumor growth or survival, while (as before) CDA monotherapy slowed tumor growth and improved survival outcomes but did not prevent most mice from succumbing to tumor growth (figure 5A, B). Co-treatment with celecoxib enhanced antitumor responses to CDA significantly, inciting rapid tumor regression and uniform survival without apparent tumor relapse until experimental end points (figure 5A, B). Moreover, no mice in this treatment group became moribund and all mice were challenged with LLC cells 60 days after initiating treatment to assess their immune status. No tumor growth was observed in mice surviving celecoxib and CDA treatment and no tumors were detected at primary lesions or at distal sites in other tissues on inspection 60 days after secondary LLC challenge (table 1, group F1). Thus, co-treatment with celecoxib and CDA induced durable protective antitumor immunity that eliminated primary tumor burdens and prevented tumor metastasis.

Since celecoxib and CDA co-treatments induced potent and robust antitumor immunity, we tested if this drug combination induced abscopal immunity that prevented growth of distal tumors not directly exposed to CDA. Accordingly, mice were inoculated to seed dermal LLC tumors (day 0) and a second bolus of LLC cells was administered 5 days later via intravenous injection to seed distal tumors in other tissues, including lungs. Celecoxib and CDA co-treatments were initiated when dermal tumors were established (as before) and 8/9 mice survived until experimental end points on day 30 (figure 5C). On inspection, no tumor growth was evident in lungs or other tissues of the eight surviving mice (table 1, group F2), indicating that celecoxib and CDA co-treatment stimulated abscopal immunity that targeted distal tumors not exposed directly to CDA, as well as primary dermal tumors exposed to CDA. Collectively, our findings reveal that CDA-induced COX2 poses a major barrier to eliciting protective antitumor responses after direct STING activation in the TME, as inhibiting COX2 led to fully protective antitumor responses to CDA that prevented growth of primary tumors and incited abscopal immunity to prevent growth of distal tumors not directly exposed to CDA.

**Figure 6:**
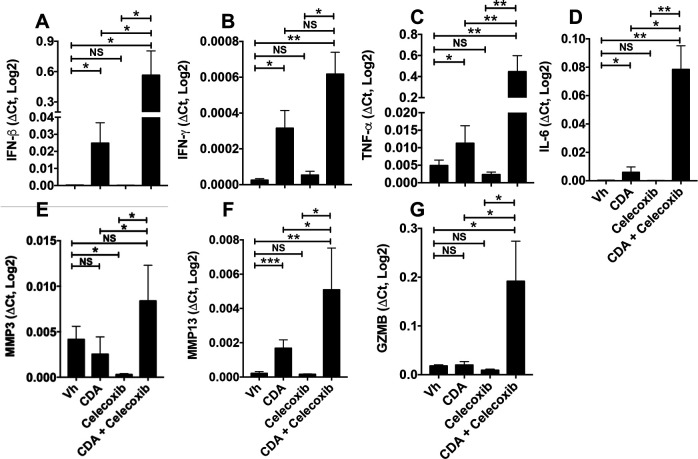
Celecoxib potentiates cyclic diadenyl monophosphate (CDA)-induced antitumor immunity. PCR analysis of gene expression in tumor lesions from mice bearing dermal Lewis lung carcinoma (LLC) tumors 3 hours after the second CDA treatment (as in figure 5). Data (mean±SEM) were analyzed using Mann-Whitney U tests, n=4–7. *P<0.05, **p<0.01, ***p<0.001. GZMB, granzyme B; IFN, interferon; IL, interleukin; MMP, matrix metalloproteinase; NS, not significant; TNF, tumor necrosis factor; Vh, vehicle.

To investigate how celecoxib impacts immune responses following STING activation, we analyzed expression of immune response genes in tumor lesions from CDA-treated mice with established LLC tumors. As expected, CDA treatment stimulated local transcription of genes encoding pro-inflammatory cytokines, IFN-β, IFN-γ, tumor necrosis factor (TNF)-α and interleukin (IL)-6 (figure 6A–D). Celecoxib co-treatment potentiated CDA-induced cytokine gene transcription, eliciting 10-fold to 40-fold increase in IFN-β, TNF-α and IL-6 transcription, relative to CDA monotherapy, while celecoxib monotherapy had no effect on basal cytokine gene transcription levels. Celecoxib also potentiated CDA-induced increase in matrix metallopeptidase 3 and 13 (MMP3, MMP13) transcription (figure 6E, F). Moreover, granzyme B (GZMB) transcription was elevated in tumor lesions given CDA and celecoxib co-treatments but was not elevated after CDA monotherapy (figure 6G), suggesting that activated effector T cells expressing GZMB accumulated in tumor lesions only if celecoxib was combined with late CDA treatment.

**Figure 7:**
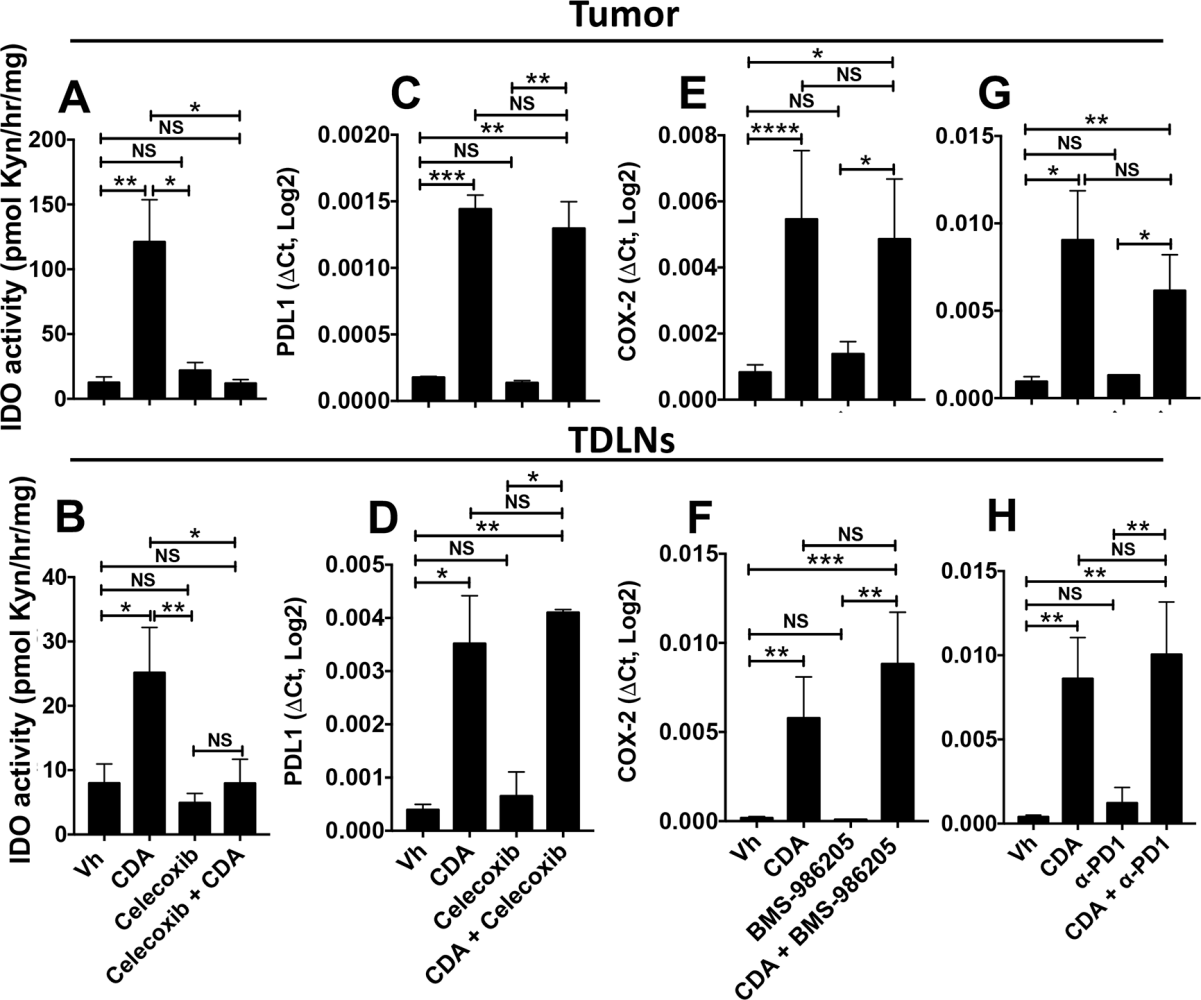
Celecoxib blocks indoleamine 2,3 dioxygenase (IDO) but not programmed death ligand-1 (PD-L1) induction and programmed death-1 (PD-1) or IDO blockade does not prevent cyclooxygenase-2 (COX2) induction after cyclic diadenyl monophosphate (CDA) treatment. (A–H) B6 mice bearing Lewis lung carcinoma (LLC) tumors were treated with CDA and celecoxib (A–D), CDA and BMS-986205 (E, F) or CDA and anti-PD1 monoclonal antibodies (mAbs) (G, H). Tumor lesions and tumor-draining lymph nodes (TDLNs) were harvested 24 hours (A–B) or 3 hours (C–H) after the second administration of CDA. IDO activity (A, B), PD-L1 (C, D) or COX2 gene expression (E–H) was assessed. Data are mean±SEM. Data were analyzed using Mann-Whitney U tests, n=4–8. *P<0.05, **p<0.01, ***p<0.001, ****p<0.0001. NS, not significant; Vh, vehicle.

In summary, inhibiting COX2 activity was more effective in unleashing the antitumor potential of CDA than simultaneous disruption of the PD-1 and IDO pathways that commonly contribute to ICPs and promote therapy resistance in many patients with cancer and mouse tumor models. To assess if celecoxib impacts other STING-responsive immune regulatory pathways, we assessed PD-L1 transcription and IDO activity in tumor lesions and TDLNs after CDA treatment. Celecoxib co-treatment completely blocked CDA-induced IDO activity in tumor lesions and TDLNs (figure 7A, B), as IDO activity levels were comparable in untreated mice with tumors and in mice co-treated with celecoxib and CDA. Celecoxib did not block CDA-induced PD-L1 transcription after STING activation (figure 7C, D). Lindrostat treatment to inhibit IDO (figure 7E, F) and PD-1 blockade (figure 7G, H) had no significant impact on CDA-induced COX2 transcription in tumor lesions or TDLNs. Thus, the superior efficacy of celecoxib, relative to IDO inhibition and PD-1 blockade, may in part be due to effective disruption of the induced IDO pathway, although celecoxib may also disrupt other regulatory pathways responsive to CDA since lindrostat was not as effective as celecoxib in unleashing the full antitumor effects of CDA.

## Discussion

In this study, we show that robust resistance to the antitumor effects of STING is an adaptive feature of the TME in mice with established LLC tumors. STING activation^21^ and PD-1 blockade to disrupt ICPs^1^ are complementary strategies to stimulate innate immune cells or reactivate dysfunctional tumor-specific T cells, respectively. Both strategies were discovered and validated in mouse tumor models, prompting clinical trials. Clinical trials to test STING activator drugs are ongoing,^11^ although a provisional report from an active phase I trial (NCT03010176) cited no objective clinical responses to direct (intratumoral) administration of a proprietary STING activator (MK-1454) in patients with advanced solid tumors or lymphomas.^22^ Thus, clinical responses to STING activators may not recapitulate the high incidence of robust antitumor responses to CDA therapy observed in some mouse tumor models.^11^ Likewise, the high incidence of effective antitumor responses to PD-1 blockade observed in some mouse tumor models does not reflect clinical outcomes, as >50% of patients with melanoma and >80% of patients with NSCLC, respectively, are refractory to PD-1 blockade monotherapy.^1 3^ Disparities in the incidence of effective antitumor responses in mouse models and patients with cancer undermine the utility of mouse models as predictors of clinical responses, and prompt the need to identify models that better reflect clinical outcomes.

We selected the LLC model for this study because LLC tumors depend on intrinsic STING signaling for optimal growth because STING/IFN-I signaling elevated IDO activity in TDLN DCs that suppress antitumor immunity.^12 23^ In contrast, immunogenic B16 tumors and LLC tumors expressing neo-antigens do not depend on STING signaling for optimal growth.^12^ Based on these observations, we reasoned that local immune balance driving immune regulatory responses in the parental LLC model would attenuate the antitumor effects of direct STING agonist (CDA) treatment. Our findings show that adaptive resistance to direct CDA treatment is indeed a major barrier to inciting effective antitumor responses in mice with established LLC tumors (>250 mm^3^). Resistance to CDA therapy was not an intrinsic attribute of LLC tumors, as initiating CDA monotherapy when small LLC tumors were first detected (~100 mm^3^) led to uniformly protective responses against primary tumors and secondary LLC challenge, indicating that mice acquired stable antitumor immunity. Therapy resistance in mice with established LLC tumors was mediated by TME pathways that rapidly increased in potency following CDA treatment. Independent co-treatments to disrupt three distinct immune regulatory pathways increased tumor control and enhanced survival significantly following CDA treatment. Thus, adaptive resistance to CDA therapy increased as LLC tumors matured, reflecting higher resistance to immunotherapy in patients diagnosed with advanced cancers.

While PD-1 blockade monotherapy did not promote antitumor responses, PD-1 blockade synergized with CDA to reduce therapy resistance and promote uniform survival. However, surviving mice did not clear primary tumor burdens, as all mice harbored invasive residual tumors emanating from primary tumor lesions, and some mice had metastatic tumors. Two proprietary IDO inhibitor drugs with distinct pharmacological characteristics,^10^ 1MT and navoximod (NLG-919), had little or no antitumor effects as monotherapies, and did not improve outcomes following CDA treatment. A third proprietary IDO inhibitor drug, lindrostat (BMS-986205), with a superior pharmacological profile, reduced resistance to CDA therapy significantly, leading to uniform survival. However, as for co-treatments with CDA and PD-1 blockade, uniform tumor relapse manifested in all surviving mice co-treated with CDA and lindrostat. These outcomes reflect observations of patient resistance to PD-1 blockade and IDO inhibitor therapy in NSCLC and other cancers,^24–26^ and testify to the key roles of the PD-1 and IDO pathways in promoting resistance to CDA therapy. Disrupting either pathway led to significant survival benefits, indicating that these pathways were not redundant but simultaneous disruption of both pathways did not further improve CDA-induced antitumor responses. Residual or resurgent activity of PD-1 or IDO pathways, or other immune regulatory pathways following CDA therapy may explain why primary tumor burdens were not eliminated.

Metabolic adaptations, including elevated IDO activity, emerged as correlative markers of poor survival of patients with cancer with advanced melanoma and renal cell carcinoma following nivolumab treatments to block PD-1 interactions.^6^ Elevated serum IDO activity also associated strongly with poor survival of patients with NSCLC following RT/CT.^7 8^ IDO-mediated resistance to ICP blockade was also described in mouse tumor models.^27 28^ Nevertheless, several IDO inhibitor drugs tested in clinical trials did not lead to significant survival benefit for patients with cancer, even when the proprietary IDO inhibitor epacadostat was administered in combination with pembrolizumab to block PD-1 interactions in the ECHO-301 phase III clinical trial.^9^ Our findings in the LLC model confirm that IDO activity dampens the antitumor attributes of direct STING activation, and testify to the technical difficulty of sustaining pharmacological IDO inhibition to overcome therapy resistance in the TME.

The COX2-specific inhibitor celecoxib was more effective in disrupting adaptive resistance to CDA therapy than PD-1 blockade or lindrostat co-treatments, even if these co-treatments were combined. Mice co-treated with CDA and celecoxib survived uniformly and acquired systemic antitumor immunity that protected mice from growth of distal LLC tumors not exposed directly to CDA (abscopal effects) and acquired long-term (memory) immunity that prevented growth of LLC tumors after secondary challenge. These outcomes revealed that the COX2 pathway is a major factor contributing to therapy resistance, and that inhibiting COX2 activity restored the full range of antitumor responses to CDA monotherapy observed in mice with smaller B16 tumors^11^ and LLC (this study) tumors. This finding is consistent with previous reports that COX2, and the ensuing prostaglandin cascade, promote carcinogenesis and suppress antitumor immunity, potentially by attenuating IFN signaling in the TME.^29–31^ Celecoxib and the non-specific COX1/2 inhibitor aspirin reduced cancer risk but did not slow growth of immunogenic tumors in mice.^29 30^ Aspirin boosted antitumor responses to PD-1 blockade but synergistic effects were modest, even though treatment was initiated only 3 days after tumor engraftment.^30^ COX2 inhibitors also slowed LLC tumor growth,^13 14^ although effects were modest, despite administering drug continuously starting 4 days after tumor engraftment.^13^ In the current study, celecoxib monotherapy had no survival benefit in mice with established LLC tumors, despite its potent synergistic effects when combined with CDA treatment. Celecoxib potentiated CDA-induced expression of genes associated with inflammation and immunity, including cytokines, extracellular matrix remodeling enzymes and GZMB expressed by effector T cells. Since STING-deficient mice bearing STING-sufficient LLC tumor cells were refractory to the antitumor effects of CDA, tumor-associated cells, not tumor cells, are the source of STING-induced IFN-β that drives antitumor responses. The ability of celecoxib to boost inflammatory and immune responses appears paradoxical, as the anti-inflammatory attributes of COX2 inhibitors are well known. A potential reason why celecoxib enhanced inflammatory and immune responses to CDA is that celecoxib blocked local increase in CDA-induced IDO activity, a potent suppressor of innate and adaptive immunity.^15^ This finding is consistent with previous reports that COX2-specific inhibitors blocked IDO induction in acute myeloid leukemia cells and in mice with LLC tumors.^14 32^ However, contrasting outcomes following co-treatments with CDA and celecoxib or lindrostat imply that IDO blockade only partially explains the superior antitumor effects of celecoxib. As IFNs stimulate PD-L1 and IDO expression, it was surprising that elevated IFN production following CDA/celecoxib co-treatment did not upregulate these regulatory pathways. However, factors that modulate PD-L1 expression and IDO activity in distinct cell types are poorly defined. For example, inhibiting the COX2/prostaglandin pathway reduced PD-L1 expression in tumor-associated macrophages (TAMs) and myeloid-derived suppressor cells in mice with MBT2 bladder carcinomas^33^ and induced TAMs to adopt an inflammatory M1 phenotype in mice with APC^Min^ intestinal tumors.^34^

In clinical settings, the requirement for direct CDA administration into tumors may be challenging, although improved image-guided drug delivery makes this approach increasingly feasible. Some STING activators may be effective when given systemically. 5,6-Dimethylxanthenone-4-acetic acid (DMXAA), which activates murine but not human STING, blocked growth of small LLC tumors when given via the intraperitoneal route, although (as for CDA) DMXAA was less effective in controlling growth of larger LLC tumors and survival was not reported.^35^ Intravenous treatment with a synthetic derivative of the natural STING activator cyclic guanyl-adenyl monophosphate (cGAMP), made by the cytosolic DNA sensor cGAMP synthase, slowed dermal CT-26 adenocarcinoma growth and enhanced mouse survival.^36^ However, these outcomes were achieved only at the highest dose tested (20 mg/kg) and dosing was initiated at CT-26 engraftment, a clinically impractical dosing regimen. In this study, survival assessments were curtailed prematurely, as 40% of vehicle-treated mice survived until experimental end points. Another study using dimerized amidobenzimidazole (diABZI) reagents to activate STING controlled CT-26 tumor growth and promoted survival when administered intravenously, although diABZI treatment was initiated just 2 days after CT-26 engraftment.^37^ Thus, STING activators may have considerable potential as antitumor immune adjuvants but use of clinically unfeasible treatments and outcome measures lacking rigor make it difficult to estimate the likely efficacy of STING activators in patients.

In summary, adaptive therapy resistance is a major barrier to achieving effective antitumor responses following direct activation of STING, particularly when the TME is mature. Our findings suggest that STING agonist monotherapy may not provoke durable and abscopal antitumor immunity in settings of clinical cancer, unless treatment is given directly to relatively small tumors. Our findings in the LLC tumor model testify to the remarkable physiological plasticity of the mature TME, and identify potential strategies to reduce therapy resistance by targeting STING-responsive pathways that suppress antitumor immunity and inflammation. For these reasons, the LLC model offers novel insights into robust barriers that preclude successful clinical immunotherapy, as mice with established LLC tumors reflect high resistance to immunotherapy in patients diagnosed with advanced cancers.

## Methods

### Mice

C57BL/6 (B6) mice were bred in a barrier facility at the Comparative Biology Centre, Newcastle University. STING-KO mice (fully backcrossed to B6 background) were described previously.^38^

### Tumor models

LLC (ATCC) tumor cells were injected intradermally (2×10^5^ cells/mouse) into the right flank of female B6 mice and tumor growth was monitored. Tumor sizes were calculated using the formula V=(d1×d2)^3/2^×(π/6), where d1 and d2 are perpendicular tumor diameters. Mice were treated with mixed linkage CDA ammonium salt (ML RR-S2 CDA or AKA ADU-S100; Insight Biotechnology, 100 µg/mouse, intratumorally or intravenously) or vehicle (deionized water), as described previously.^11^ Some mice were treated with celecoxib (Sigma-Aldrich, 60 mg/kg, oral gavage), 1MT (2 mg/mL, drinking water), navoximod (NLG-919, 5 mg/kg, intraperitoneal), kindly provided by NewLink Genetics, BMS-986205 (2 mg/kg, oral gavage), kindly provided by Bristol-Myers Squibb, anti-mPD1 (Clone RMP1-14, BioXcell), anti-IFNAR (MAR1-5A3), IgG2a isotype (clone 2A3, BioXcell) or IgG1 isotype control (MOPC-21, BioXcell) mAbs (150 µg/injection, 15 min before each CDA injection). Vehicles for BMS, navoximod and celecoxib were 5% dimethyl sulfoxide/10% Tween 20/30% polyethylene glycol/55% phosphate-buffered saline (PBS) (added in this order). 1MT was dissolved in 0.1 M NaOH, pH adjusted to 7.4 with 0.1 M HCl, 10 tablets/l of Hermesetas (Sodium Saccharin, Sucralose; Carrier: L-Leucine) sweetener added, and volume adjusted with water to give 2 mg/mL. To evaluate abscopal effects, mice were injected with 2×10^5^ cells/mouse (intradermally) and 5 days later with 5×10^5^ cells/mouse (intravenously).

### IDO enzyme activity

IDO activity was measured in TDLN or tumor lesions as described previously.^16^ In brief, tissues were homogenized in PBS, added to IDO enzyme cocktails and kynurenine generated after 2 hours was measured by high-performance liquid chromatography.

### Quantitative RT-PCR

RNA was extracted using Tri reagent (Sigma-Aldrich), using the manufacturer’s recommended procedure. RNA was then reverse-transcribed using a random hexamer cDNA RT kit (Clontech), and quantitative RT-PCR was performed using SsoFast EvaGreen supermix (Bio-Rad). PCR primers (murine) were as follows (5’−3’, forward and reverse);

β-actin: TACGGATGTCAACGTCACAC & AAGAGCTATGAGCTGCCTGA;

COX2: ATCATAAGCGAGGACCTGGG & CTGCAGGTTCTCAGGGATGT;

PD-L1: TGCGGACTACAAGCGAATCA & CTTCTCTTCCCACTCACGGG;

Granzyme B: GAAGCCAGGAGATGTGTGCT & GCACGTTTGGTCTTTGGGTC;

MMP3: CTATACGAGGGCACGAGGAG & CCACCCTTGAGTCAACACCT

MMP13: GGAGCCCTGATGTTTCCCAT & ATCAAGGGATAGGGCTGGGT;

TNF-α: TCGTAGCAAACCACCAAGTG & GGAGTAGACAAGGTACAACC

IL-6: AGACAAAGCCAAGT CCTTCAGAGA & GCCACTCCTTCTGTGACTCCAGC

IFN-γ: CCTTCTTCAGCAACAGCAAGGCG & CCCACCCCGAATCAGCAGCG

IFN-β1: GCAGCTGAATGGAAAGATCA & GTGGAGAGCAGTTGAGGACA

Threshold cycle (Ct) values were set in the early linear phase of amplification; relative expression of target genes were calculated as 2Ct(β-actin)−Ct(target gene).

### Statistical analysis

Data were analyzed using GraphPad Prism. For tumor growth, two-way analysis of variance with Bonferroni post hoc multicomparison tests were performed. Unpaired two-tailed non-parametric Mann-Whitney U tests were performed for two group comparisons. Statistical significance for survival curves was evaluated using the log-rank test.
