# Supplementary material for: Overcoming resistance to STING agonist therapy to incite durable protective antitumor immunity
Source: J Immunother Cancer. 2020 Aug 26;8(2):e001182. doi: 10.1136/jitc-2020-001182 (PMC7451475; doi:10.1136/jitc-2020-001182)
Supplement: Supplementary data [file jitc-2020-001182supp001.pdf]

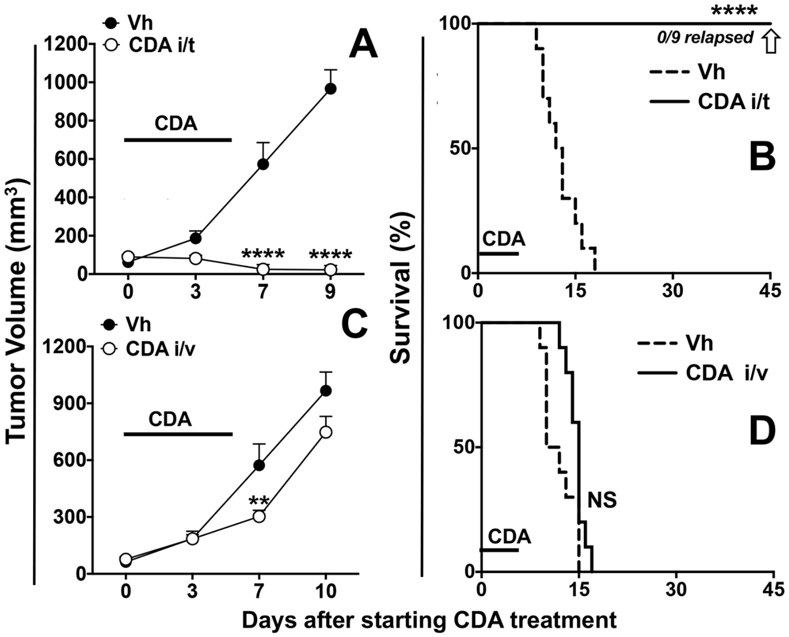

Supplemental Figure S1. Direct CDA treatment when LLC tumors first appear protects mice. B6 mice were treated with CDA (100mg/mouse, i/t or i/v, days 0, 2, 6) when LLC tumors first appeared (90-120mm^3). Tumor volumes (AC) and mouse survival (BD) were scored until experimental endpoints (day 45). At endpoints all mice were examined to assess tumor clearance or relapse. Data (mean ± SEM) were analyzed using two-way ANOVA with Bonferroni's multiple comparisons test for each time point (AC) or Log-rank test (BD). N=9-10. **p<0.01, ***p<0.001 ****p<0.0001, NS, not significant.

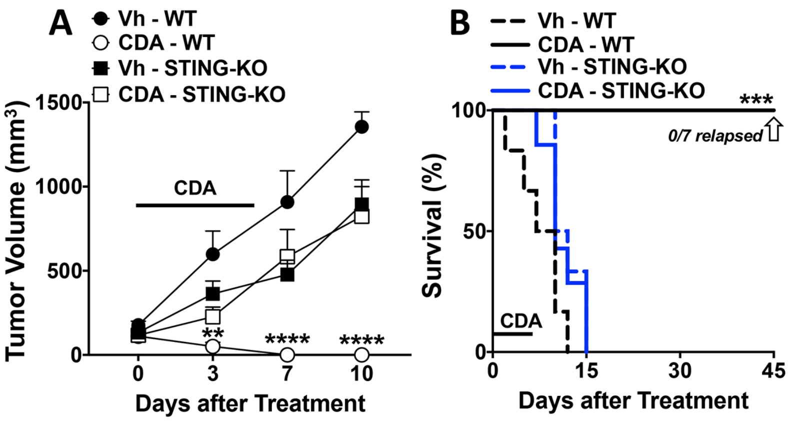

Supplemental Figure S2. CDA promotes anti-tumor responses by targeting STING in accessory cells not LLC tumor cells. LLC tumors were grown in B6 and STING-KO mice and treated with CDA (100mg/mouse, i/t, days 0, 2, 6) starting when small tumors first appeared (<120mm^3). Tumor volumes and survival were scored until experimental endpoints. At endpoints all mice were examined to assess tumor clearance or relapse. Data (mean ± SEM) were analyzed using two-way ANOVA with Bonferroni's multiple comparisons test for each time point (A), or Log-rank test (B). N=6-7. **p<0.01, ****p<0.0001 (vs vehicle).

Lemos H, *et al. J Immunother Cancer* 2020; 8:e001182. doi: 10.1136/jitc-2020-001182

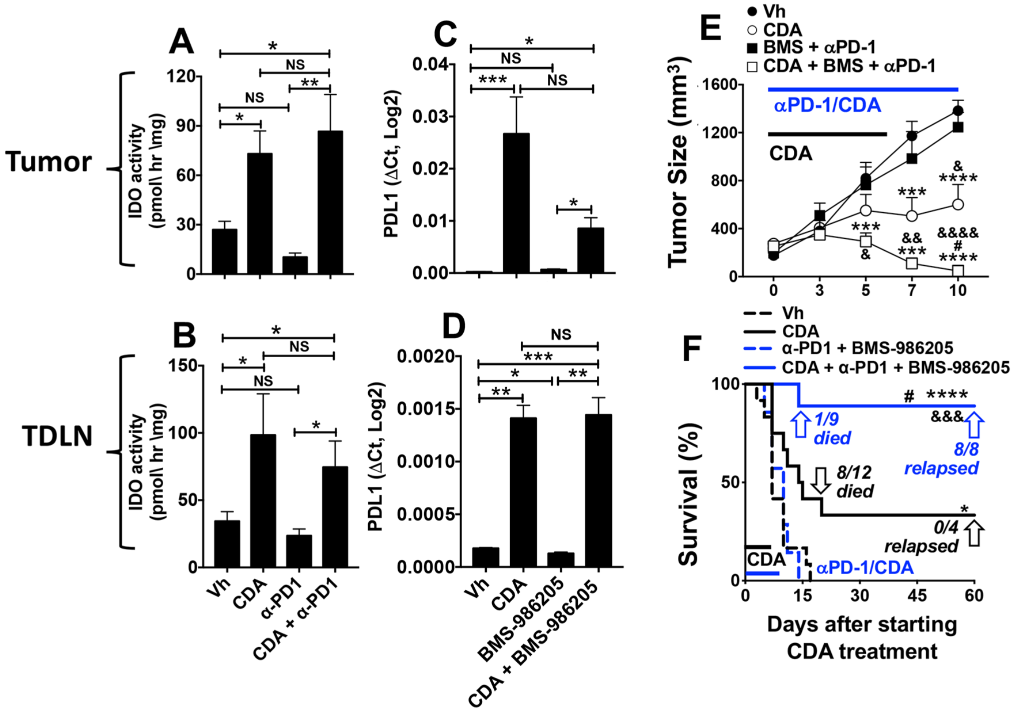

Supplemental Figure S3. STING activation enhances the PD-1 and IDO pathways independently. A-D. PD-L1 expression (AB) and IDO activity (CD) were assessed in tumor lesions (AC) and TDLNs (BD) of B6 mice with LLC tumors treated with CDA/BMS and CDA/anti-PD1 mAbs as in Figures 2 & 3, respectively. Tissues were harvested 3h (for qPCR) or 24h (for IDO activity) after the second CDA treatment (day 2). **EF**. Tumor growth and survival of mice treated with CDA, anti-PD-1 mAbs and IDO inhibitor (BMS-986205). At endpoints all mice were examined to assess tumor clearance or relapse. Data (mean ± SEM) were analyzed using Mann-Whitney tests (A, B, C, D), or two-way ANOVA with Bonferroni's multiple comparisons test for each time point (E), or Log-rank test (F). A-D, N=5-8; E-F, N=7-12. *p<0.05, **p<0.01, ***p<0.001, ****p<0.0001 (vs vehicle); #p<0.05 (vs CDA monotherapy); &p<0.05, &&p<0.01, &&& p<0.001, &&&&p< 0.0001 (vs anti-PD1 + BMS986205 monotherapy).

Lemos H, *et al. J Immunother Cancer* 2020; 8:e001182. doi: 10.1136/jitc-2020-001182
